# Signaling defenses with color: a meta‐analysis of leaf color variation, palatability, and herbivore damage

**DOI:** 10.1111/nph.70243

**Published:** 2025-05-27

**Authors:** Tatiana Cornelissen, Fernando A. O. Silveira, Susan Vieira Gomes, Xosé Lopez‐Goldar, Sylvie Martin‐Eberhardt, William Wetzel

**Affiliations:** ^1^ Center for Ecological Synthesis and Conservation Universidade Federal de Minas Gerais Belo Horizonte Minas Gerais 31270‐901 Brazil; ^2^ Department of Integrative Biology Michigan State University East Lansing MI 48824 USA; ^3^ Department of Land Resources and Environmental Science Montana State University Bozeman MT 59717‐3120 USA; ^4^ Department of Ecology and Evolutionary Biology Cornell University Ithaca NY 14853 USA; ^5^ School of Biological Sciences Illinois State University Normal IL 61761 USA; ^6^ Department of Plant Biology Michigan State University East Lansing MI 48824 USA

**Keywords:** delayed greening, herbivory, leaf color, leaf color polymorphism, red leaves

## Abstract

- We investigated the impact of leaf color variation on herbivory, testing current hypotheses indicating that leaf color could influence herbivory through bottom‐up control (by signaling leaf quality and defenses) or top‐down control (by attracting predators).
- A comprehensive phylogenetic meta‐analysis was conducted to assess the effects of leaf color on defense traits, leaf palatability, herbivore fitness, and herbivory.
- We show that nongreen leaves were better defended, less nutritive, and experienced less herbivory, leading to a reduction in herbivore fitness. Stronger effects of leaf color on herbivory were found in tropical plants, whereas lowered leaf quality in nongreen leaves was found in temperate plants. Increased leaf defense and reduction in insect fitness traits were observed in both temperate and tropical nongreen leaves.
- Our results indicate that leaf color plays a significant role in shaping plant defenses, leaf nutritive value, and herbivore fitness, ultimately modulating levels of herbivory. This suggests coordination between leaf color, defenses, and quality, which may be responsible for patterns of variation in herbivory and fitness‐related traits in herbivores.

## Introduction

A complex interplay of leaf functional traits such as size (Zhu *et al*., 2024), specific leaf area (Poorter *et al*., 2004; Kozlov *et al*., 2015), shape (Ferris, 2019; Higuchi & Kawakita, 2019), nutrient stoichiometry (Njovu *et al*., 2019; Schön *et al*., 2023), and mechanical and chemical defenses (Hanley *et al*., 2007; Caldwell *et al*., 2016; Agrawal *et al*., 2021) determines how herbivores find and use resources. Leaf palatability, therefore, reflects a syndrome of coordinated leaf traits that ultimately shape plant resistance and/or tolerance to insect herbivory, which in turn is countered by insect adaptation, driving the coevolutionary dynamics between insects and host plants (Ehrlich & Raven, 1964; Archetti *et al*., 2009; Agrawal & Zhang, 2021). Despite overwhelming evidence of the effects of plant interspecific trait variability on herbivory (see Wetzel *et al*., 2023; Liu *et al*., 2024; Zvereva *et al*., 2024), considerably little attention has been paid to intraspecific leaf color variability that occurs between plant populations or within individual plants. The intraspecific diversity in leaf size, shape, morphology, and color is typically suggested as the product of selective pressures optimizing trait combinations to cope with abiotic conditions that drive strategies of resource use, acquisition, and conservation (Campitelli *et al*., 2008; Hughes *et al*., 2022). However, leaf functional traits also influence how plants interact with insect herbivores, but which leaf traits influence variation in herbivory and how these variations arise and are selected have been a long‐lasting debate in plant–herbivore interactions (Harper, 1977; Carmona *et al*., 2011; Muiruri *et al*., 2019).

While the role of color has been extensively studied in animals, particularly in the contexts of color vision, aposematism, and sexual selection (see Wiens & Emberts, 2024), a comprehensive understanding from the plant perspective is emerging more recently (but see Landi *et al*., 2015; Renoult *et al*., 2017). Research has predominantly focused on flower and fruit color, a key trait mediating interactions with pollinators, frugivores, and seed dispersers. However, the significance of leaf color and its implications for understanding plant–herbivore interactions and plant defense mechanisms remain largely underexplored. Leaf color variability is commonly observed in phylogenetically unrelated species and across biogeographic regions (e.g. Lee & Collins, 2001; Lee, 2002; Gong *et al*., 2020; Hughes & Lev‐Yadun, 2023), with nonphotosynthetic pigments arguably playing dual roles in plant physiology and defense (Archetti *et al*., 2009). The diverse range of leaf color polymorphisms not only likely reflects a complex interplay between environmental factors and physiological processes associated with photoprotection and photoinhibition (Gould *et al*., 2002; Karageorgou *et al*., 2008; Archetti *et al*., 2009; Menzies *et al*., 2016) but also other sources of stress such as heavy metals (Landi, 2015), temperature (Renner & Zohner, 2019), and nutrient deficiency (Liang & He, 2018). Still, leaf polymorphism may also indicate the end product of evolutionary pressures driven by leaf herbivores (Archetti, 2009; Cooney *et al*., 2012; Menzies *et al*., 2016; Lev‐Yadun, 2023). Although valuable insights about the ecological role of leaf coloration have been accrued over the past 20 yr (Archetti, 2000; Hamilton & Brown, 2001; Lev‐Yadun, 2006, 2014, 2016, 2022; Lev‐Yadun & Gould, 2007, 2009; Archetti *et al*., 2009), our understanding of its significance in mediating plant–herbivore interactions has been mostly focused on autumn leaves. The lack of synthetic views on how leaf color influences herbivory prevents us from generating insights into the adaptive strategies employed by plants to deter or tolerate herbivory under different environmental conditions.

Ontogenetic and seasonal leaf color change is usually coordinated with leaf traits shown to affect herbivory, in which the contribution of mechanical features (Caldwell *et al*., 2016) and chemical compounds in shaping herbivore selective feeding and ovipositing sites is widely recognized (Moore *et al*., 2014; Richards *et al*., 2015; Wetzel & Whitehead, 2020; Muller & Junker, 2022). Variation in color of leaves, petioles, and stems (Lev‐Yadun *et al*., 2004; Lev‐Yadun, 2016) can occur during ontogeny, under varying physiological conditions and seasonality in both temperate and tropical regions (Kursar & Coley, 1992; Archetti, 2009; Queenborough *et al*., 2013). For example, leaf color is linked to plant defenses against herbivores, representing a covariate of trait syndromes associated with the accumulation of secondary compounds (see Cooney *et al*., 2012) and mechanical defenses (e.g. toughness and trichome density; Poorter *et al*., 2004; Hughes *et al*., 2022). These traits often co‐vary during leaf ontogeny, as seen in nongreen leaves during young, early developmental stages, which later transition to harder, green, and more herbivore‐prone leaves (Chen & Huang, 2013; Ochoa‐López *et al*., 2015; Dayrell *et al*., 2018).

**Fig. 1:**
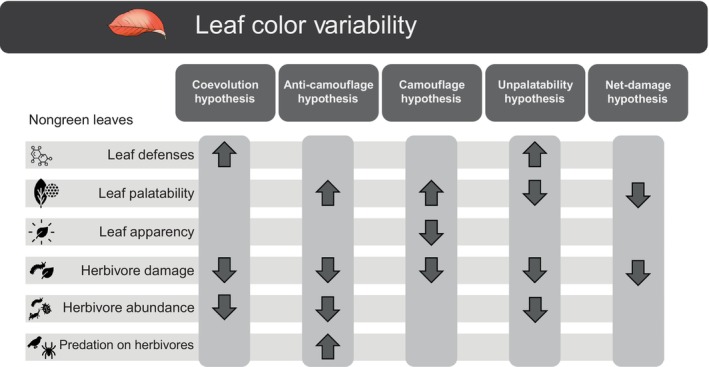
Conceptual models and predictions of the effects of leaf color on herbivory based on classical hypotheses aiming to explain intra‐ and interspecific variation in plant traits, including leaf defense and leaf quality, and insect traits associated with fitness. The response variables commonly evoked by authors to explain differences in herbivory levels in nongreen leaves are listed on the left. Arrows indicate either an increase (pointed up) or a decrease (pointed down) of such variables in nongreen, colored leaves.

Several hypotheses have been proposed to explain the relationship between leaf color variability and herbivory incidence and intensity (e.g. Givnish, 1990), as summarized in Fig. 1. The coevolutionary or signaling hypothesis (Archetti, 2000; Hamilton & Brown, 2001; Archetti & Brown, 2004) suggests that nongreen leaves represent honest warning signs of low leaf palatability due to higher concentration and diversity of defensive compounds such as secondary metabolites so that lower herbivory of such leaves would benefit both herbivores and plants. The camouflage hypothesis (Stone, 1979; Karageorgou & Manetas, 2006; Niu *et al*., 2017, 2018) suggests that nongreen leaves might not be perceived by insects and would therefore escape herbivory. The camouflage hypothesis pertains specifically to red leaves and animals that do not perceive red wavelengths, such as most mammals and many insects (Döring *et al*., 2009; Hughes *et al*., 2021; Van Der Kooi *et al*., 2021). The anti‐camouflage hypothesis (or the undermining insect camouflage; Lev‐Yadun *et al*., 2004) proposes that nongreen leaves (especially young red leaves) would enhance the conspicuousness of insect herbivores, making them more apparent and more vulnerable to visually oriented predators, resulting in low herbivory levels. The unpalatability hypothesis (Coley & Aide, 1989; Archetti, 2009) suggests that low herbivory in nongreen leaves or plants with nongreen leaves is due to the presence of nongreen pigments that act as herbivore deterrents, benefiting plants by a direct gustatory effect (Schoonhoven, 1969; Van Loon, 1990). Finally, the net‐damage hypothesis (Kursar & Coley, 1992), initially proposed for tropical plants with delayed greening, poses that nongreen leaves are both less nutritive and visually attractive to herbivores, which may function to delay herbivory until the leaves are better mechanically protected. While temperate plants also produce nongreen young leaves, the adaptive significance of this trait may differ from tropical delayed greening, as it often co‐occurs with abiotic stress or variegation (e.g. Gould *et al*., 2002; Hughes & Lev‐Yadun, 2023). Although these five hypotheses differ mechanistically, they are functionally similar in that all lead to lower levels of herbivory, due to either bottom‐up control via leaf quality or to top‐down control via predators or parasites (Fig. 1).

Although hypotheses addressing the role of leaf color as an important driver of plant–herbivore interactions have been discussed and exposed to criticism (e.g. Lev‐Yadun, 2006) over the past 25 yr (see Archetti, 2000, 2009; Lev‐Yadun, 2003, 2016, 2022; Hughes & Lev‐Yadun, 2023), a quantitative synthesis incorporating the predictions of the main hypotheses accounting for leaf color variability and its effects on herbivory is still lacking. Here, we aimed to integrate the current hypotheses relating the adaptive value of leaf color with a deep and comprehensive examination of how leaf color influences herbivory patterns. To this end, we conducted a meta‐analysis that incorporates the effects of plant phylogeny and biogeographic region to provide a quantitative assessment of the effects of color on (1) plant traits associated with leaf defense and leaf palatability or nutritional quality, (2) traits associated with the fitness of insect herbivores, and (3) interactions between herbivorous insects and plants via leaf consumption. We tested the following predictions: (1) Nongreen leaves will show lower herbivory than green leaves, with stronger effects in tropical species where delayed greening is a common anti‐herbivore strategy; (2) Nongreen leaves will exhibit higher defense traits, with stronger effects in tropical plants due to stronger selection pressure from herbivores on young leaves; (3) Nongreen leaves will have lower nutritional quality, with temperate plants showing stronger reductions due to the prevalence of stress‐ or senescence‐related pigments (e.g. anthocyanins in aging leaves); and (4) Herbivore fitness will be lower on nongreen leaves across regions, reflecting their dual role as defended (tropics) or low‐quality (temperate) resources.

## Materials and Methods

### Literature search

The literature search was conducted in the Web of Science Core Collection and Scopus, using search terms commonly indexed in the literature on leaf color. Online searching was conducted as of February 2024, covering the entire time span of each search engine (1945–2024), and keywords were used as independent strings encompassing aspects of leaf traits, insect traits, and herbivory in the literature on leaf color. Keyword selection was supplemented using the litsearch package (Grames *et al*., 2019) on R (R Core Team, 2024) and keyword co‐occurrence was checked and clustered using VosViewer (Van Eck & Waltman, 2014). These combined approaches generated 24 keywords (Supporting Information Methods S1); studies were only searched with English keywords.

The studies from this initial literature search (*n* = 1486) were screened by title, abstract, full text, and evaluated for eligibility following PRISMA guidelines (Page *et al*., 2020; O'Dea *et al*., 2021). Studies were included in the final database if they addressed the effects of leaf color in plant–insect interactions, evaluating quantitative aspects of leaf quality, leaf defense, herbivore fitness, and herbivory levels, in both green and nongreen leaves of angiosperms. We only considered studies comparing green and nongreen leaves within the same species, either between individuals (e.g. color morphs) or within individuals (e.g. ontogenetic shifts). Studies comparing communities of species with exclusively green or nongreen leaves were excluded. After two full‐text screenings, 27 studies met our inclusion criteria (PRISMA flowchart, Methods S1).

### Database assembly

From these 27 studies, we gathered information on: (1) authorship, publication date, country and region (temperate, including Mediterranean, or tropical) of the study; (2) biome, host plant species, botanical family, and leaf color (red, purple, pink, white, or yellow as nongreen leaves); and (3) broad herbivore group (caterpillars, beetles, grasshoppers, or aphids) and guild (chewers or sap‐feeders). We also recorded how variability in leaf color was evaluated in those plants (between individual plants or plant populations; within individual plants) nested with the mechanisms (leaf color polymorphism, leaf variegation, delayed greening, differences in leaf color margin, and autumn colors) that generated leaf color variability. Several response variables were evaluated in these studies, and we focused our review on plant, insect, and interaction response variables associated with herbivory that enabled the computation of effect sizes (Figs S1, S2).

Response variables were grouped into leaf defenses (including secondary compounds such as tannins, polyphenols, trichome density, anthocyanin content, carbon and leaf toughness), leaf nutrients or nutritional quality (Chl and nitrogen content, specific leaf area, and 1/(C : N) ratio), proxies for herbivore fitness (growth rate, development rate, fecundity, biomass, and survival), and herbivory, which encompassed measurements of herbivory incidence (frequency of attacked leaves, frequency of feeding scars), herbivory intensity (proportion of leaf area removed), and herbivore abundance (number of herbivores feeding upon leaves). We did not include herbivory on leaves fallen to the ground because data are not readily available (Heinrich & Collins, 1983). To be included in the set of response variables, studies had to clearly report means, measurements of variation (SE, SD, confidence intervals (CI)), and sample size of the response variable(s) in both green and nongreen leaves of the same plant species. These 27 studies generated 160 effect sizes, as 88% addressed more than one response variable (Table 1). Response mean values (*X*_green_, *X*_nongreen_), SD (SD_green_, SD_nongreen_), and sample size (*N*_green_, *N*_nongreen_) for each response were gathered from the text, tables, and/or figures. Data from the figures was extracted using ImageJ (Schneider *et al*., 2012).

**Table 1:** List of the 27 studies used in meta‐analysis evaluating the effects of leaf color on herbivory, leaf nutritional quality, leaf defenses, and insect traits associated with fitness.

Study	Author and year	Plant species studied	Mechanism of leaf color variation	Scale of study	Region	Response variables
1	Agrawal & Spiller (2004)	*Conocarpus erectus*	Leaf color polymorphism	BTW	Tropical	Herbivory, leaf defenses, leaf quality
2	Ayestaran & Alcala (2016)	*Pinguicula moranensis*	Leaf color polymorphism	BTW	Temperate	Herbivory
3	Baisden *et al*. (2018)	*Rhus copallinum* *Viburnum dentatum* *Cornus florida* *Cornus sericea* *Liquidambar styraciflua*	Leaf color polymorphism	BTW	Temperate	Herbivory
4	Ballas & Matter (2020)	*Sedum lanceolatum*	Leaf color polymorphism	BTW	Temperate	Herbivory, leaf defenses
5	Brennan & Weinbaum (2001)	*Eucalyptus pilularis*	Young nongreen adult green	WTN	Temperate	Herbivory
6	Campitelli *et al*. (2008)	*Hydrophyllum virginianum*	Leaf variegation	BTW	Temperate	Herbivory, leaf quality
7	Cooney *et al*. (2012)	*Pseudowintera colorata*	Leaf margin	BTW	Temperate	Herbivory, leaf defenses
8	Costa‐Arbulu *et al*. (2001)	*Sorghum halepense*	Leaf color polymorphism	WTN	Temperate	Insect traits
9	Döring *et al*. (2009)	*Prunus padus*	Leaf color polymorphism	WTN	Temperate	Herbivory
10	Farnier *et al*. (2014)	*Eucalyptus camaldulensis* *Eucalyptus kitsoniana*	Young nongreen adult green	WTN	Temperate	Herbivory, leaf defenses, leaf quality
11	Farnier & Steinbauer (2016)	*Eucalyptus camaldulensis* *Eucalyptus kitsoniana*	Young nongreen adult green	WTN	Tropical	Herbivory
12	Gerchman *et al*. (2012)	*Brassica oleracea* *Salvia viridis*	Leaf color polymorphism	WTN	Tropical	Herbivory, leaf defenses
13	Gomes & Cornelissen (2025)	*Lecythis pisonis*	Young nongreen adult green	WTN	Tropical	Herbivory, leaf quality
14	Gould *et al*. (1995)	*Begonia pavonina* *Triolena hirsuta*	Leaf color polymorphism	BTW	Tropical	Leaf defenses, leaf quality
15	Hughes *et al*. (2010)	Veronica spp. (5 species)	Leaf margin	BTW	Temperate	Herbivory, leaf defenses
16	Ide (2022)	*Perilla frutescens* *Ipomoea batatas*	Leaf color polymorphism	BTW	Temperate	Herbivory, leaf quality, insect traits
17	Karageorgou & Manetas (2006)	*Quercus coccifera*	Young nongreen adult green	WTN	Temperate	Herbivory
18	Kursar & Coley (1992)	*Ouratea lucens* *Connarus semidecandrus* *Xylopia macrantha* *Desmopsis panamensis* *Annona spraguei*	Young nongreen adult green	WTN	Tropical	Herbivory, leaf quality
19	Markwick *et al*. (2012)	*Malus pumila*	Leaf color polymorphism	BTW	Temperate	Leaf defenses, insect traits
20	Menzies *et al*. (2016)	*P. colorata*	Leaf color polymorphism	BTW	Temperate	Herbivory, leaf defenses, leaf quality
21	Mercader *et al*. (2007)	*Fraxinus cuspidata* *Liriodendron tulipifera* *Prunus serotina*	Leaf color autumn	WTN	Temperate	Herbivory, insect traits
22	Numata *et al*. (2004)	*Nolana acuminata* *Shorea macroptera* *Shorea multiflora* *Shorea ovalis*	Leaf color polymorphism	BTW	Tropical	Herbivory
23	Portillo‐Nava *et al*. (2021)	*Amaranthus hybridus*	Leaf color polymorphism	BTW	Tropical	Herbivory, leaf defenses, insect traits
24	Sadof *et al*. (2003)	*Plectranthus scutellarioides*	Leaf variegation	BTW	Temperate	Insect traits, leaf quality
25	Schaefer & Rolshausen (2007)	*Sorbus aucuparia*	Leaf color polymorphism	BTW	Temperate	Herbivory
26	Sulifoa *et al*. (2015)	*Abelmoschus manihot*	Leaf color polymorphism	BTW	Tropical	Herbivory
27	Vogado *et al*. (2022)	*Brachychiton acerifolius* *Phaleria clerodendron*	Young nongreen adult green	BTW	Tropical	Leaf quality

All studies included in the meta‐analysis compared green vs nongreen leaves within plant species. Refer to the main manuscript for a description of categories of studies (BTW, between individual plants; WTN, within individual plants) of the same species (e.g. BTW, red vs green morphs within a polymorphic species; WTN, young red vs mature green leaves on the same plant). Complete citations are listed in the Reference list and indicated by an asterisk (*).

### Meta‐analysis

To address the effects of leaf color on (1) leaf traits, (2) insect traits, and (3) herbivory, we used the standardized mean difference between green and nongreen leaves from each individual study to calculate individual values of Hedge's *g*. Because our study focused on intraspecific variability on leaf color and herbivory, the meta‐analysis did not include studies that compared community‐wide variation in leaf color (e.g. Chen & Huang, 2013; Gong *et al*., 2020). Because red leaves were the most common color leaves in our database, we grouped all color combinations (e.g. purple, pink, and red leaf margins) into a larger category called ‘Red Leaves’. Yellow and silver leaves were kept separately. For each plant species, green leaves were assigned to the control group, and nongreen leaves were assigned to the treatment group. Individual Hedge's *g* was calculated for each register (*n* = 160), and the cumulative overall Hedge's *g* was calculated using a weighting method with the reciprocal of the sampling variances (Koricheva *et al*., 2013). Negative Hedge's *g* values indicate a decrease in the response variable in nongreen leaves, and positive values indicate an increase in the response variable. Effect sizes were calculated using the *escalc* function on metafor (Viechtbauer, 2010) and mean effects are considered significant when confidence intervals do not overlap with zero (Borenstein *et al*., 2011). Plots of mean effect sizes, prediction intervals (which show heterogeneity among effect sizes), and individual effect sizes scaled by their precision (inverse of the SE) were conducted using the orchard 2.0 package (Nakagawa *et al*., 2023).

To examine the relationship between leaf color and phylogenetic relatedness, we first reconstructed the phylogenetic tree of the studied plant species using v.phylomaker2 (Jin & Qian, 2022) and for the sake of clarity, the only gymnosperm species in our database (*Juniperus virginiana* in Baisden *et al*., 2018) was removed from the reconstructed tree. Species names were checked and updated using the R package lcvplants (Freiberg *et al*., 2020), and the six species absent from the Open Tree Taxonomy super phylogeny tree were bound to their designated congeneric species. For all plant species, we calculated evolutionary distances using the rotl package (Michonneau *et al*., 2016). We then reconstructed the plant phylogeny for each dataset (each one of the four response variables), calculating a covariance matrix using the phytools package (Revell, 2024). The covariance matrix of distances was added as a random variable in each model.

We first ran an overall random model for each response variable, including three levels, to account for nonindependence among studies (level 1), nonindependence among registers or outcomes within the same study (level 2), and plant phylogeny (level 3). Models were calculated for each response variable using the maximum likelihood estimator and *rma* function (Viechtbauer, 2010; Gao & Carmel, 2020) and model goodness of fit was evaluated using Akaike information criterion corrected for small sample sizes. In addition, we ran mixed‐model multilevel meta‐analyses in which we incorporated moderators of the effects of leaf color on leaf and insect traits and herbivory. Due to the predictions of the five hypotheses (Fig. 1) associated with the adaptive value of leaf color, the moderators of effect sizes included in the models were region (tropical or temperate) and insect guilds (chewers or sapsuckers). We also tested whether the mechanism responsible for leaf color variability influenced the strength of the mean effects of leaf color on herbivory.

### Publication bias

The total heterogeneity of effect sizes in all models was evaluated using the *Q*‐statistic, partitioning heterogeneity between groups (levels on moderators) and within groups (error), using a chi‐square distribution with *n* − 1 degrees of freedom, where *n* equals the number of comparisons in each model. We also computed the heterogeneity statistic *I* ^2^, which ranges from 0 to 1 (low = 25%, medium = 50%, and high = 75%), and indicates the proportion of variance not due to sampling error variance (Nakagawa *et al*., 2017). The robustness of the meta‐analysis was also evaluated through funnel plots, the calculation of Rosenthal's fail‐safe numbers (Koricheva *et al*., 2013), and Egger's regression (Table S1).

## Results

**Fig. 2:**
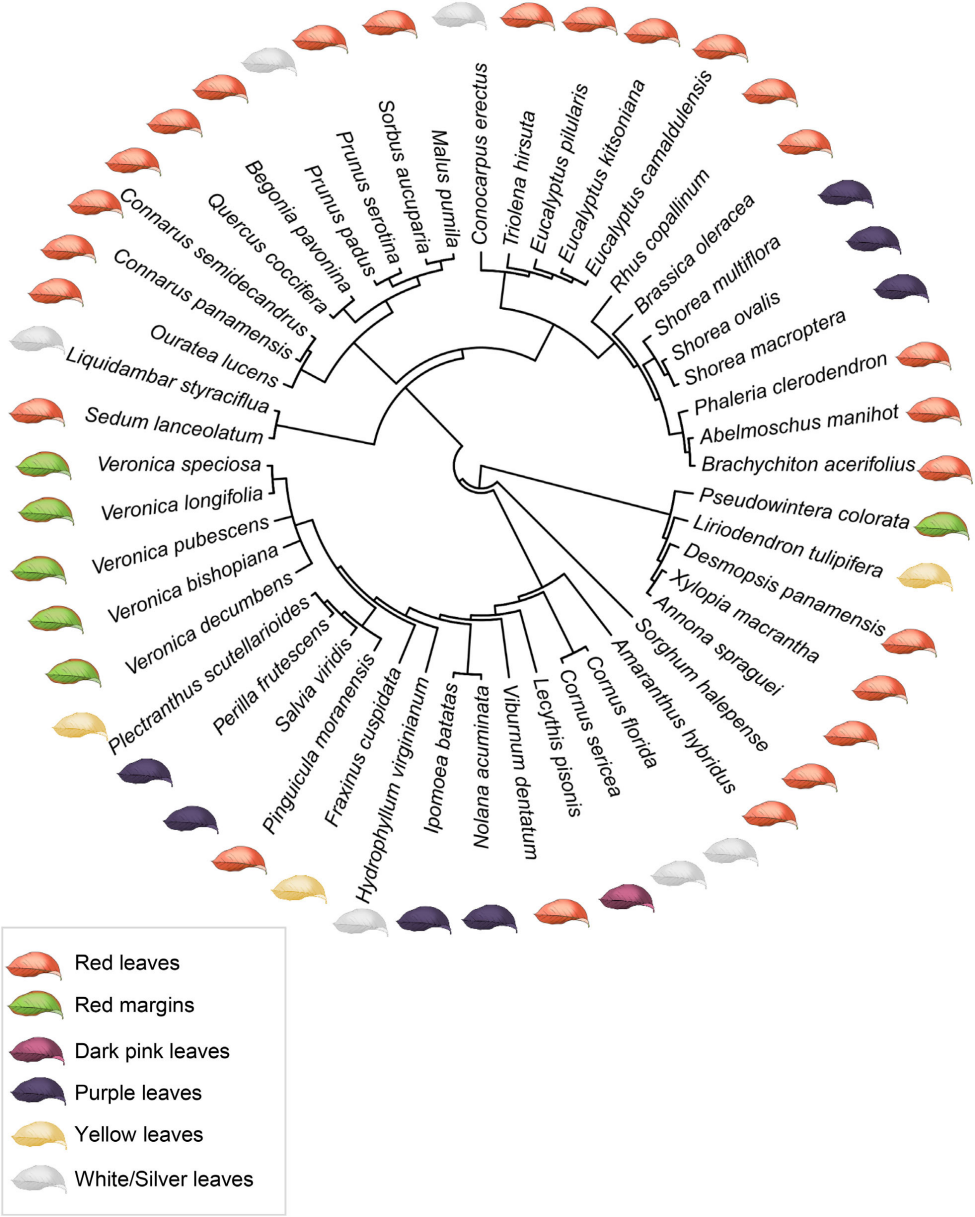
Phylogenetic reconstruction of plant species used in the meta‐analysis evaluating the effects of nongreen leaves on herbivory, leaf defenses, leaf quality, and insect traits associated with fitness. Icons denote the nongreen leaf type (e.g. red, purple, white) or pattern (e.g. green leaves with red margins) compared to green leaves within the same species.

Studies evaluating the intraspecific effects of leaf color on herbivory that met our inclusion criteria were conducted over the past 30 yr in 18 different countries, in both tropical (12 studies, 63 effect sizes) and temperate regions (15 studies, 97 effect sizes). Twenty‐seven studies of 47 plant species (Fig. 2) from 29 families resulted in a total of 160 effect sizes. Most effect sizes (59%) included paired comparisons of green and red leaves, with fewer comparing green with purple (14.4%), white/silver (13.8%), red margins (7.5%), and yellow (3.1%). More details on our qualitative results can be found in Notes S1.

**Fig. 3:**
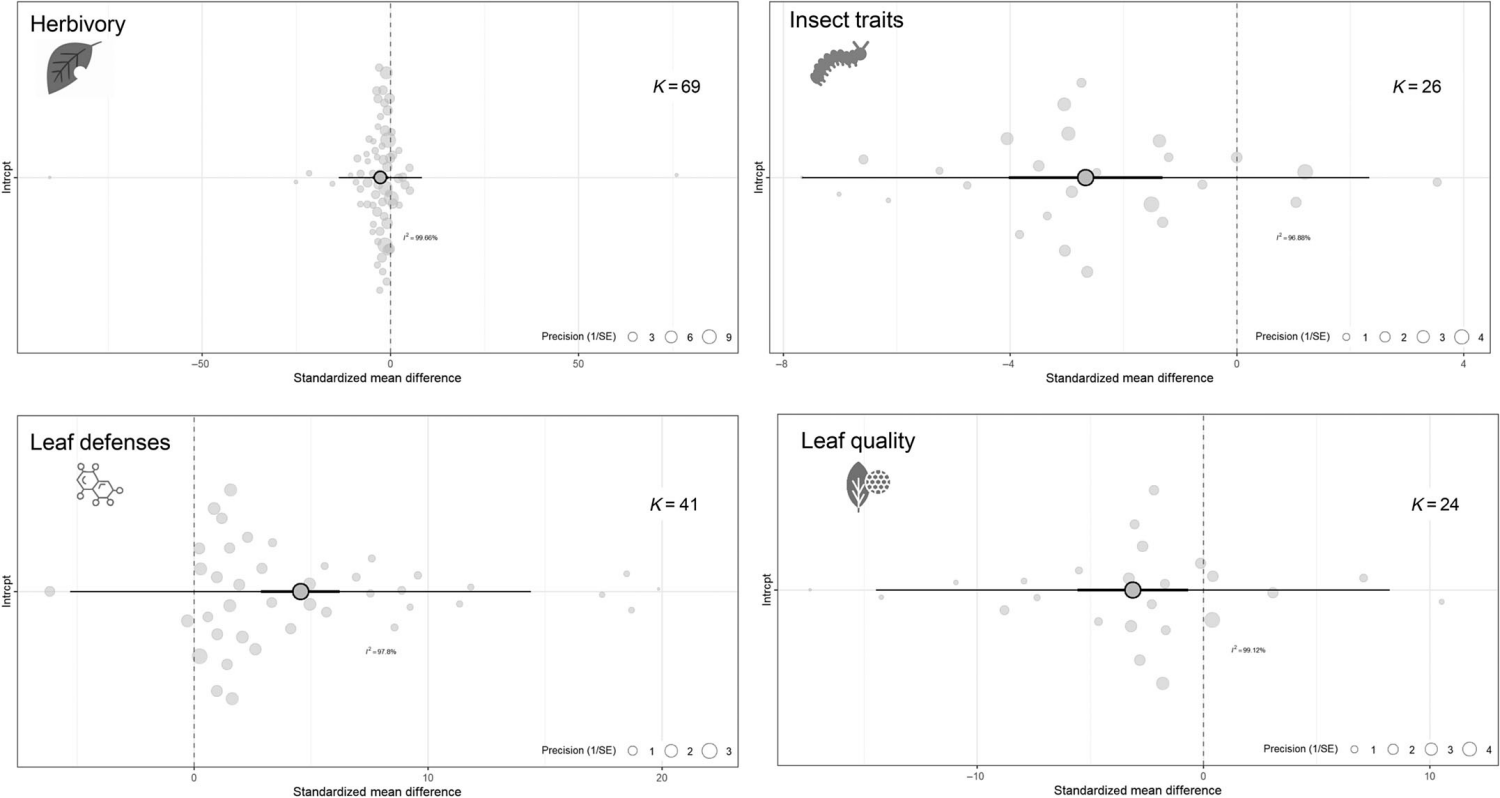
Overall effect sizes of leaf color on response variables associated with herbivory, herbivore fitness, leaf defenses, and leaf quality in plant species with nongreen leaves. Orchard plots indicate the mean overall effect with its associated 95% confidence interval (thick horizontal lines), the prediction confidence intervals around effect sizes (slim horizontal lines), and individual effect sizes weighed by their precision (1/SE). An overlap between confidence intervals and the dashed vertical lines indicates nonsignificant effects (*k* = number of independent comparisons).

We found strong evidence for the effects of leaf color variation on leaf traits, insect performance, and herbivory (Fig. 3). Overall, compared to green leaves, nongreen leaves exhibited higher defensive traits (*g* = 4.55, SE = 0.85, CI = 2.87–6.23), lower nutritional quality (*g* = −3.11, SE = 1.24, CI = −5.56 to −0.66), experienced less herbivory (*g* = −2.61, SE = 1.06, CI = −4.7 to −0.51), and their consumption reduced insect performance (*g* = −2.66, SE = 0.69, CI = −4.02 to −1.3). Overall, phylogenetic models indicated that plant phylogenetic distance significantly influenced cumulative effect size for leaf defenses (estimate = 15.62, df = 18, *P* < 0.05), but did not influence leaf quality effect sizes (estimate = 0.00, df = 17, *P* = 0.264), herbivory effect sizes (estimate = 0.00, df = 35, *P* = 0.41), or insect fitness (estimate = 1.85, df = 7, *P* = 0.084).

**Fig. 4:**
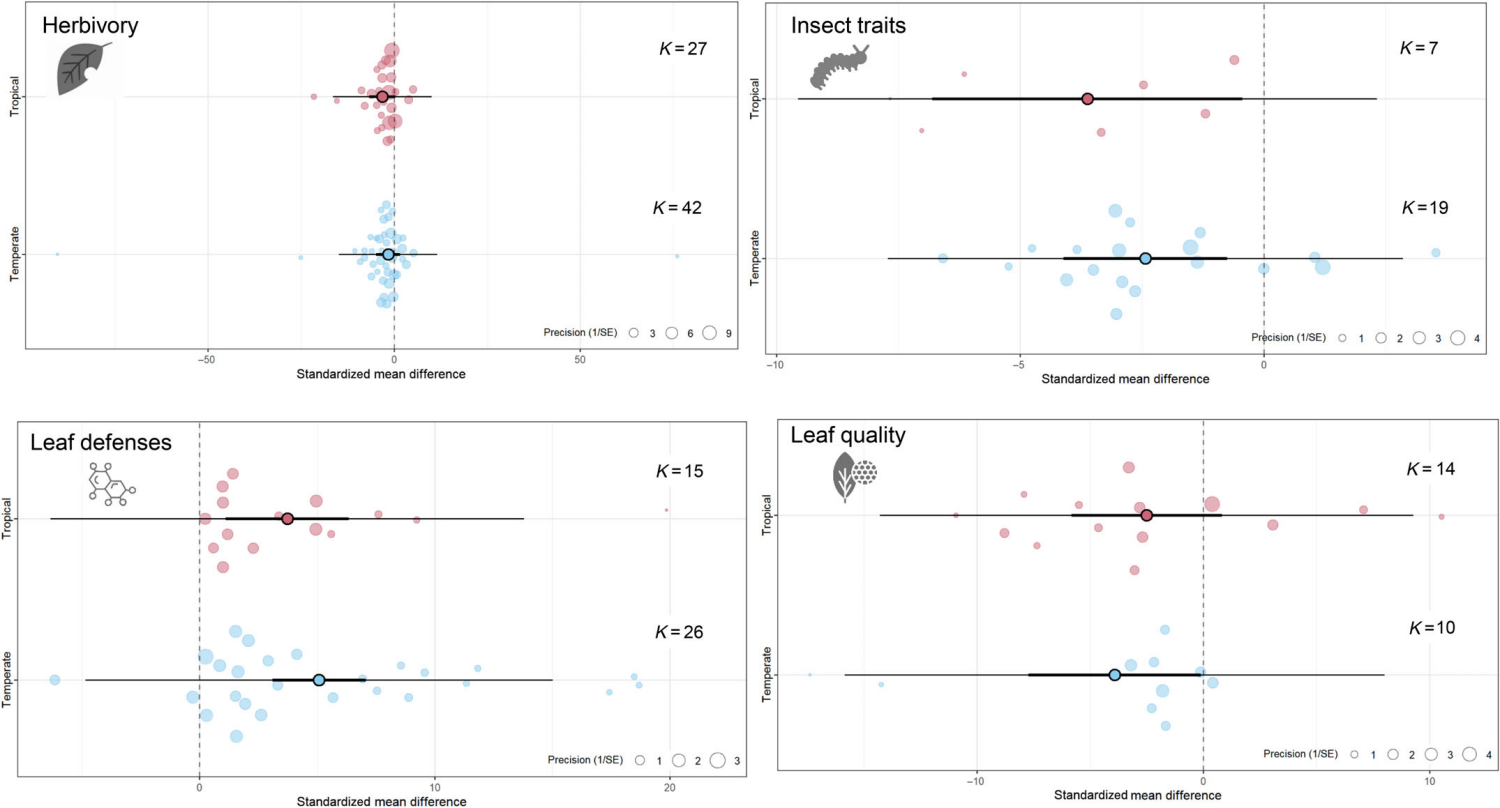
Effects sizes of leaf color on response variables associated with herbivory, insect traits associated with fitness, leaf defenses, and leaf quality in tropical and temperate regions. Orchard plots indicate the mean overall effect with its associated 95% confidence interval (thick horizontal lines), the prediction confidence intervals around effect sizes (slim horizontal lines), and individual effect sizes weighed by their precision (1/SE). An overlap between confidence intervals and the dashed vertical lines indicates nonsignificant effects (*k* = number of independent comparisons).

The strength of the effects of leaf color was modulated in most cases by latitude (Fig. 4). Nongreen leaves showed higher defenses in temperate (*g* = 5.08, CI = 3.09–7.07) than in tropical species (*g* = 3.72, CI = 1.11–6.34), although not significantly different (*Q*_B_ = 0.051, df = 1, *P* = 0.820). Lower leaf quality in nongreen leaves was mainly driven by temperate plants (*g* = −3.91, CI = −7.73 to −0.10) with no significant effect of leaf color on leaf quality in tropical plants (*g* = −2.49, CI = −5.82 to 0.82; *Q*_w_ = 47.39, df = 13, *P* < 0.001). Stronger effects of leaf color on herbivory were found for tropical compared to temperate plants (*g* = −3.19, *g* = −1.62, respectively) and consumption of nongreen leaves significantly reduced herbivore fitness (*Q*_B_ = 8.05, df = 1, *P* = 0.0045) in both tropical (*g* = −3.61, CI = −6.8 to −0.43) and temperate (*g* = −2.42, CI = −4.1 to −0.70) regions.

Herbivore guild also influenced the patterns of attack on leaves of different color patterns, but overall reductions in herbivory in nongreen leaves were detected only for chewers (*g* = −2.20, CI = −2.78 to −1.63, *n* = 59), with no significant effect on sap‐feeders such as aphids and mealybugs (*g* = −0.03, CI = −1.45 to 1.38, *n* = 10). When the guild was nested into latitude, reduced herbivory by sap‐feeders on colored leaves was detected only in tropical plants (*g* = −4.24, CI = −7.97 to −0.51, *n* = 4). For the other response variables (leaf defenses, leaf quality, and insect traits), the low sample size and representation of sap‐feeders in the database impaired the inclusion of guild as a moderator of the effect sizes.

There was no detectable effect of the scale at which the study was conducted (between individuals *g* = −2.11, within individuals *g* = −2.89) on herbivory levels (*Q*_B_ = 0.0062, df = 1, *P* = 0.93). When mechanisms generating leaf color variability were evaluated, stronger effects of leaf color on herbivory were observed for leaf color polymorphisms (*g* = −3.15, CI = −3.85 to −2.44, *n* = 29) and delayed greening (*g* = −2.12, CI = −1.92 to −0.31, *n* = 21). Other groups such as autumn leaf color, colored margins of the leaves, and leaf variegation had small sample sizes that did not enable analyses.

### Assessment of publication bias

Heterogeneity was high for all response variables evaluated, and *I* ^2^ values ranged between 96.88 and 99.65, with a larger contribution of variation arising from individual studies compared to individual outcomes. Fail‐safe numbers were high (> 5*k* + 10, where *k* is the number of comparisons) in all models, indicating the robustness of our results. Trim and fill analyses for each response variable (Fig. S3) indicated that a small number of studies would need to be included in the analyses to turn funnel plots into a symmetrical shape, also indicating the robustness of the results found.

## Discussion

Herbivorous insects use chemical and visual cues to locate, identify, and consume suitable host plants and/or plant parts (Bruce, 2015; Richards *et al*., 2015; Blande, 2017). Our meta‐analytical results demonstrate that leaf color significantly modulates herbivory levels, with nongreen leaves experiencing lower herbivory across both tropical and temperate regions. Crucially, our analysis of effect sizes reveals this pattern is most strongly associated with two factors: significantly higher chemical defenses (Hedge's *g* = 4.55) and lower nutritional quality (Hedge's *g* = −3.11) in nongreen leaves. While other mechanisms like camouflage or mechanical defenses have been proposed (e.g. Lev‐Yadun *et al*., 2004; Karageorgou & Manetas, 2006), our dataset contained insufficient studies measuring these variables to test their effects statistically. The consistent reduction in herbivore fitness and lower herbivory by chewers further supports that chemical/nutritional traits drive these patterns, as chewers are particularly sensitive to such leaf properties (Caldwell *et al*., 2016).

Our results support the general hypothesis that leaf color represents a trait syndrome associated with visual, chemical, and mechanical defenses that influence herbivory incidence and intensity. Specifically, our findings support both the coevolutionary and unpalatability hypotheses, but with limited support for the palatability hypothesis, as leaf palatability measured in terms of leaf quality was lower in nongreen leaves. The role of color mediating tri‐trophic interactions via herbivore predation has also been suggested in the anti‐camouflage hypothesis (Lev‐Yadun *et al*., 2004), which proposes that nongreen leaves undermine herbivore camouflage, making insects more visible to predators and thus reducing herbivory indirectly by deterring colonization (see Lev‐Yadun *et al*., 2004). However, this hypothesis inherently limits its own testability: If nongreen leaves effectively prevent herbivore establishment, few studies would record herbivore presence or predation rates on such leaves, which may aid in explaining the scarcity of quantitative data in our meta‐analysis. Future experimental work – manipulating predator access to herbivores on green vs nongreen leaves or the use of artificial prey – is needed to explicitly test this mechanism (see Koski *et al*., 2017). Most hypotheses relating color polymorphisms to variation in leaf herbivory rely on the assumption that nongreen color is associated with leaf traits that might indicate resource quality (Hypotheses 2, 4, and 5 in Fig. 1) such as the concentration of secondary compounds such as anthocyanins, which are honest signals to herbivores in red, purple, or pink leaves. Red leaves (e.g. *Sedum lanceolatum*; Ballas & Matter, 2020) or leaves with red margins (e.g. *Pseudowintera colorata*; Cooney *et al*., 2012) might serve as visual cues signaling leaf unpalatability, which can be detected by several herbivores known to recognize such hues (Briscoe & Chittka, 2001; Chen *et al*., 2021). The red color is usually a warning signal of the defensive chemical status of leaves to potential insect herbivores, and although this is not explicitly claimed in all hypotheses, all hypotheses assume that leaf quality is important for herbivore selection and feeding, and that direct or indirect leaf defenses would reduce herbivory in such leaves.

Color is correlated with leaf properties that influence herbivore loads and ultimately herbivory (see Archetti *et al*., 2009; Lev‐Yadun, 2016, 2023; Ide, 2022; Hughes & Lev‐Yadun, 2023). Our findings suggest that nongreen leaves are associated with reduced herbivory, with stronger effects in tropical plants. This aligns with the coevolutionary hypothesis, which proposes that plants have evolved to develop red leaves as a defense mechanism against herbivores. The stronger effect of leaf color on reducing herbivory in the tropics could be attributed to the higher diversity and specialization of herbivores in these regions (Kursar & Coley, 1992; Becerra, 2015), leading to a more pronounced coevolutionary response between plants and herbivores compared to temperate regions. In our phylogenetic meta‐analysis, we assessed the variations in leaf color at the species level, either within or between individual plants. Concurrently, community‐level herbivory data corroborate our overall finding of lower leaf quality mediating lower herbivory in nongreen leaves (Coley & Aide, 1989; Chen & Huang, 2013; Gong *et al*., 2020). We examined a select number of studies carried out in tropical communities, focusing on plants producing exclusively green or red leaves, especially during their early growth stages. Our brief analysis revealed a trend of reduced herbivory in nongreen plants compared to their green counterparts (see Table S2). This summary supports the prevalent hypotheses that leaf color functions as a warning signal to herbivores, highlighting its adaptive significance. Leaf coloration could potentially serve as an adaptive defense mechanism in tropical and temperate plants, particularly when physical defenses, such as leaf toughness, are not yet fully developed in young leaves (Aide, 1992). This hypothesis is supported by studies showing increases in mechanical defenses as young red leaves mature into expanded green leaves (see Chen & Huang, 2013). Furthermore, the observed differences in defense mechanisms between plants that initially had green or red leaves tend to disappear once those leaves mature (see Gong *et al*., 2020).

The expression and maintenance of leaf coloration usually entails some metabolic costs, due to the associated costs of pigment synthesis and their reduced photosynthetic capacity (but see Hara, 1957; Shelef *et al*., 2019). The accumulation of chemical compounds in colored leaves is therefore adaptive if the benefits associated with pigment accumulation in leaves compensate for the negative effects of nonphotosynthetic pigments that might compete with Chl or are costly to plants (Lee, 2002; Archetti *et al*., 2009; Hughes & Lev‐Yadun, 2023). Anthocyanins were the single most common secondary compound evaluated in the studies reviewed here, although other compounds such as tannins, sesquiterpene polygodials, and phenolics were also common. Anthocyanins and other phenolic compounds such as tannins are usually positively correlated in red leaves, as shown for 96 tropical plant species in China (see Gong *et al*., 2020), as these two compounds share the same biosynthetic pathway (Fineblum & Rausher, 1997; Schaefer & Rolshausen, 2006; Tanaka *et al*., 2008) and may represent the main line of chemical defense in colored leaves.

Overall, our study showed that plant phylogenetic relatedness was important only for plant defenses, and nongreen leaves exhibited a strong and significant increase in chemical and mechanical defenses compared to green leaves, providing support for the coevolutionary and the unpalatability hypotheses. The fact that most studies to date involve chewer herbivores highlights the importance of understanding how the diet breadth of antagonists (specialists vs generalists) and how plant secondary chemistry influence herbivore feeding selection and resource assimilation. However, formal tests of the coevolutionary hypothesis will require addressing the mechanistic links between host plant resource use, diet breadth, and plant defenses on leaves of different colors. None of the studies reviewed here have simultaneously integrated leaf defense, leaf quality, insect traits, and herbivory intensity for a single plant–herbivore system, limiting our understanding of the adaptive mechanistic value of leaf color in both tropical and temperate plants. Studies conducted over leaf ontogeny are also necessary to understand whether shifts in leaf color that occur from young to older leaves, especially in tropical systems where delayed greening is common (Kursar & Coley, 1992), are accompanied by shifts in the way herbivores locate and use plant resources.

Autumn leaf senescence is widespread in temperate deciduous forests (Lee, 2002) and red foliage is usually a result of anthocyanin production in senescing leaves (Hoch *et al*., 2001; Gould, 2004). More recent studies suggested that leaf color is adaptive and not only a byproduct of leaf senescence (Chen & Huang, 2013), especially for species that maintain red leaves when they are young. This is the case for several unrelated plant species in the tropics (Kursar & Coley, 1992), subtropics (Lev‐Yadun *et al*., 2012; Gong *et al*., 2020), and in temperate forests (Archetti & Brown, 2004; Lev‐Yadun *et al*., 2012; Lev‐Yadun, 2016), although mechanisms usually evoked to relate leaf color and herbivory in different latitudes or different leaf ontogenies are quite different (Lev‐Yadun, 2016). The coevolutionary hypothesis (Archetti, 2000; Hamilton & Brown, 2001; Archetti & Brown, 2004) was initially proposed to explain yellow and red autumn coloration in temperate plants and its effects on herbivores. This hypothesis is rooted in the idea of coloration as a true warning sign, in which both plants and herbivores would benefit, following co‐evolutionary paths due to the benefits of reduced herbivory in plants and increased efficiency in host selection and resource feeding in insects. Our meta‐analysis revealed strong effects of leaf color on leaf traits associated with increased leaf defense in both tropical and temperate plants, which supports the idea of an honest warning sign via leaf coloration.

Both tropical and temperate plants showed higher concentrations of secondary compounds in nongreen than in green leaves. Reductions in leaf quality, measured as reduced amounts of Chl and nitrogen, were found only for temperate plants, whereas reductions in fitness‐related insect traits were found for both tropical and temperate plants. Therefore, resource advertisement in terms of leaf palatability via leaf defenses is supported for plants regardless of leaf ontogeny, as coloration of leaves due to anthocyanins is transient but expressed in both young leaves (e.g. tropical, subtropical, and temperate plants) or in old senescent leaves (e.g. in temperate plants). These patterns align with functional hypotheses: In temperate plants, nongreen leaves (often associated with senescence or abiotic stress) prioritize resource conservation (Archetti *et al*., 2009), leading to lower nutritional quality, while tropical plants leverage delayed greening primarily as an anti‐herbivore strategy, resulting in stronger herbivory reductions. Thus, leaf color polymorphisms likely serve divergent roles across latitudes – buffering stress in temperate systems and deterring herbivores in the tropics – while consistently reducing insect performance.

Lastly, the lack of significant effects on leaf quality in the tropics, despite the observed impacts on herbivory and insect traits, suggests that the relationship between leaf color and plant quality may be more complex in tropical ecosystems, such as the humid tropical forests reviewed here. This could be attributed to the more complex dynamics of plant–insect interactions (Novotny & Basset, 2005) and ecological factors unique to tropical regions, which may influence the expression of plant quality traits in response to leaf color.

The degree of leaf damage by insects is a result of multiple and complex processes, driven by how herbivores find host plants and leaves and how they deal with factors associated with leaf palatability. One of the main assumptions of the coevolution hypothesis is that herbivorous insects have the ability to recognize nongreen leaves as an honest signal of resource quality. The camouflage and the anti‐camouflage hypotheses propose that plants may use conspicuous leaf coloration as a signal to deter herbivores and/or attract their natural enemies. Contrary to previous findings (e.g. Archetti *et al*., 2009; Lev‐Yadun, 2016), our results show that the influence of nonphotosynthetic compounds on insect host encounters and feeding through increased levels of leaf defenses in nongreen leaves may serve as a mechanism to counteract herbivory, supporting the idea that leaf color can function as a visual cue or even signal against herbivores.

Overall, our findings provide valuable insights into the multifaceted relationships between leaf color, plant defenses, herbivory, and insect traits, conceptually and biogeographically extending our current knowledge regarding the prevailing hypotheses and highlighting the ecological significance of leaf color polymorphisms regardless of plant phylogeny and biogeographic region. The integration of functional traits related to plant defense into the framework of defense syndromes (Agrawal, 2020; Ruiz‐Guerra *et al*., 2020) should therefore include leaf color polymorphisms and leaf color over plant ontogeny (e.g. Massad, 2013). As an important trait influencing herbivory and related to plant species strategies shaped by evolutionary history, leaf color polymorphisms should be incorporated into the current theory of plant defense against insect herbivores.

## Competing interests

None declared.

## Author contributions

TC designed the database. SVG and TC collected data. TC, FAOS, XL‐G, SM‐E and WW contributed to data analysis, manuscript preparation, and revision. Submission was accomplished by TC.

## Disclaimer

The New Phytologist Foundation remains neutral with regard to jurisdictional claims in maps and in any institutional affiliations.

## Supporting information


**Fig. S1** Variables extracted from primary data and variable grouping.
**Fig. S2** Alluvial plot of meta‐analysis moderators.
**Fig. S3** Trim and fill funnel plots.
**Methods S1** Literature search, keyword co‐occurrence, and PRISMA flowchart.
**Notes S1** Results of qualitative data of leaf color variation among plants.
**Table S1** Assessment of publication bias.
**Table S2** Studies of leaf color variation conducted at the community scale.
Please note: Wiley is not responsible for the content or functionality of any Supporting Information supplied by the authors. Any queries (other than missing material) should be directed to the *New Phytologist* Central Office.

## Data Availability

Data available on FigShare (doi: 10.6084/m9.figshare.28945454).
