# Supplementary material for: Signaling defenses with color: a meta‐analysis of leaf color variation, palatability, and herbivore damage
Source: New Phytol. 2025 May 27;247(2):884–96. doi: 10.1111/nph.70243 (PMC12177304; doi:10.1111/nph.70243)
Supplement: Supplementary file 1 — Fig. S1 Variables extracted from primary data and variable grouping. Fig. S2 Alluvial plot of meta‐analysis moderators. Fig. S3 Trim and fill funnel plots. Methods S1 Literature search, keyword co‐occurrence, and PRISMA flowchart. Notes S1 Results of qualitative data of leaf color variation among plants. Table S1 Assessment of publication bias. Table S2 Studies of leaf color variation conducted at the community scale. Please note: Wiley is not responsible for the content or functionality of any Supporting Information supplied by the authors. Any queries (other than missing material) should be directed to the New Phytologist Central Office. [file NPH-247-884-s001.pdf]

New Phytologist Supporting Information

Article title: Signaling defences with color: a meta-analysis of leaf color variation, palatability and herbivore damage

Authors: Tatiana Cornelissen, Fernando A. O. Silveira, Susan Vieira Gomes, Xosé Lopez-Goldar, Sylvie Martin-Eberhardt, William Wetzel

Article acceptance date: 02 May 2025

The following Supporting Information is available for this article:

Methods S1

1) LITERATURE SEARCH

Keyword search was conducted in February 2022 and February 2024 on Web of Science and Scopus, using the following keywords after the implementation of keyword search in *litsearch* (Grames et al. 2019):

Topic of Search= (anthocyanin* OR varieg* OR "autumn* colour* " OR "delay* green* " OR "leaf* color* " OR "leaf* colour* " OR "red leav* " OR "visual* cues " OR "young* leaf* " OR "young* leav* " OR "autumn* leav* " OR "visual* signal* ") **AND** (folivor* OR "insect* herbivor* " OR "leaf* herbivor* " OR "specialist* herbivor* " OR "generalist* herbivor* " OR "herbivor* damag* " OR "herbivor* insect* " OR "herbivory")

2) CO-OCCURRENCE OF KEYWORDS

Network of keyword co-occurrence in initial literature search. Map was created based on bibliographic data downloaded from WoS and Scopus using the co-occurrence tool and full counting method. The minimum number of co-occurrences was set to 20, which identified a total of 167 keywords. From these, total link strength varied between 24 (e.g., color vision, mimicry) and 552 (e.g., anthocyanins, evolution) and keywords with stronger links and a minimum number of clusters at 5 are plotted.

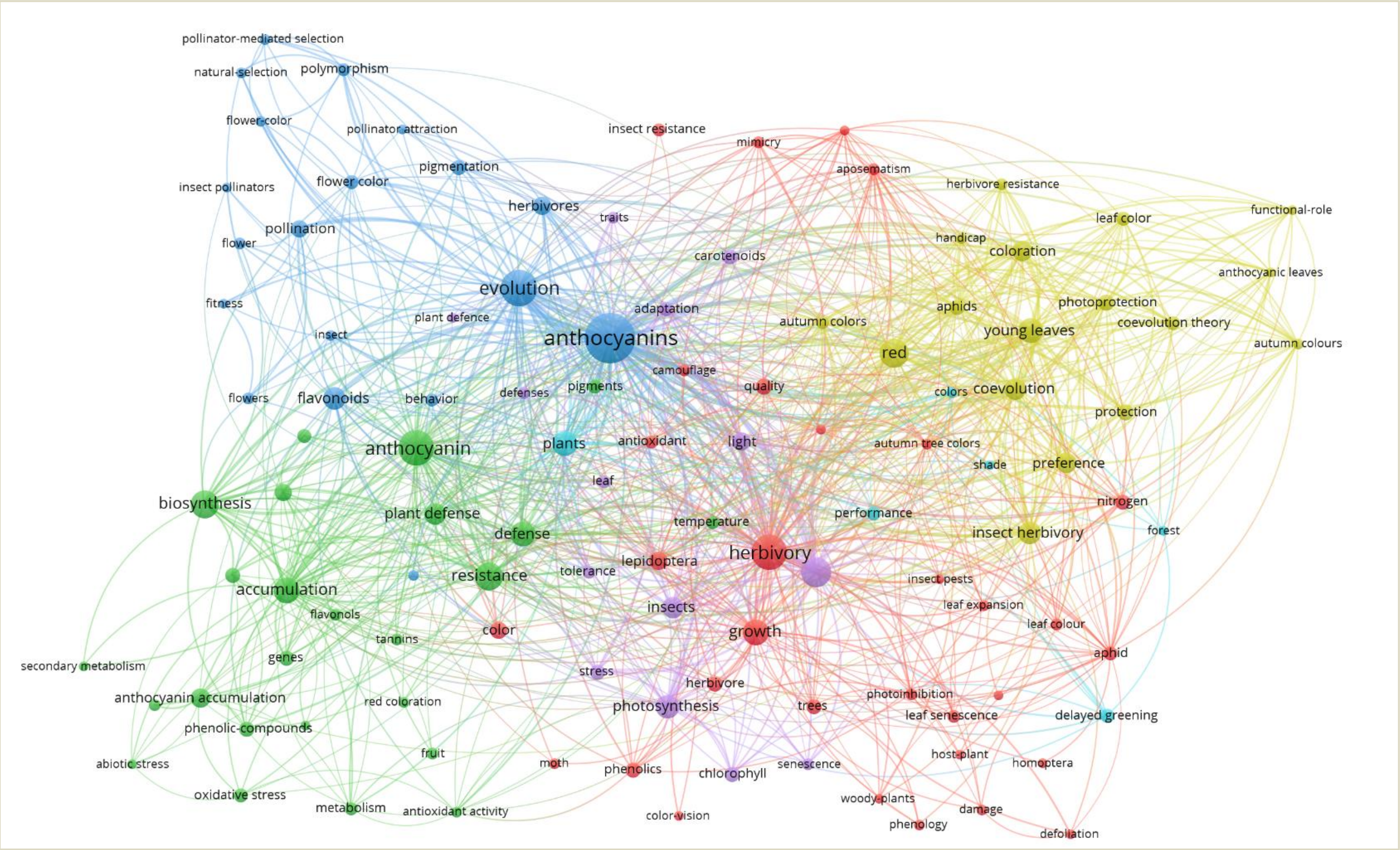

3) PRISMA FLOWCHART (following guidelines in Page et al. 2021, O'Dea et al. 2021)

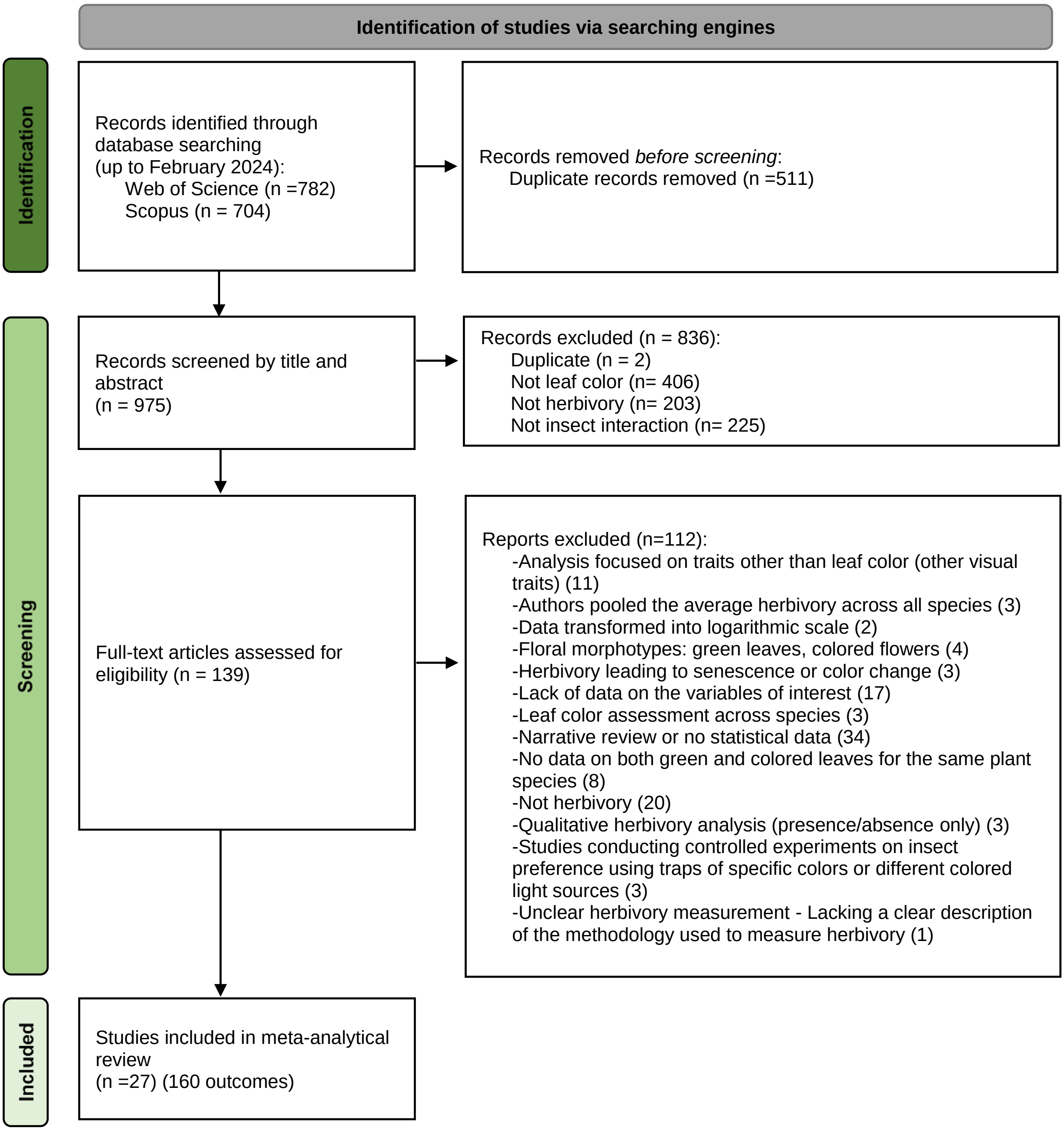

New Phytologist

Fig. S1

Variables extracted from primary data and variable grouping. **A)** Scale of study (between or within plants with different leaf colors) and mechanisms responsible for variation in leaf color; **B)** Response variables reported in the primary literature and grouping of such responses into four broad response variables (SLA= specific leaf area, CN ratio = Carbon: Nitrogen ratio, Herbv= Herbivory, GR= growth rate, DT=development time). Size of circles is proportional to the amount of comparisons in each category of response variables extracted from the primary literature.

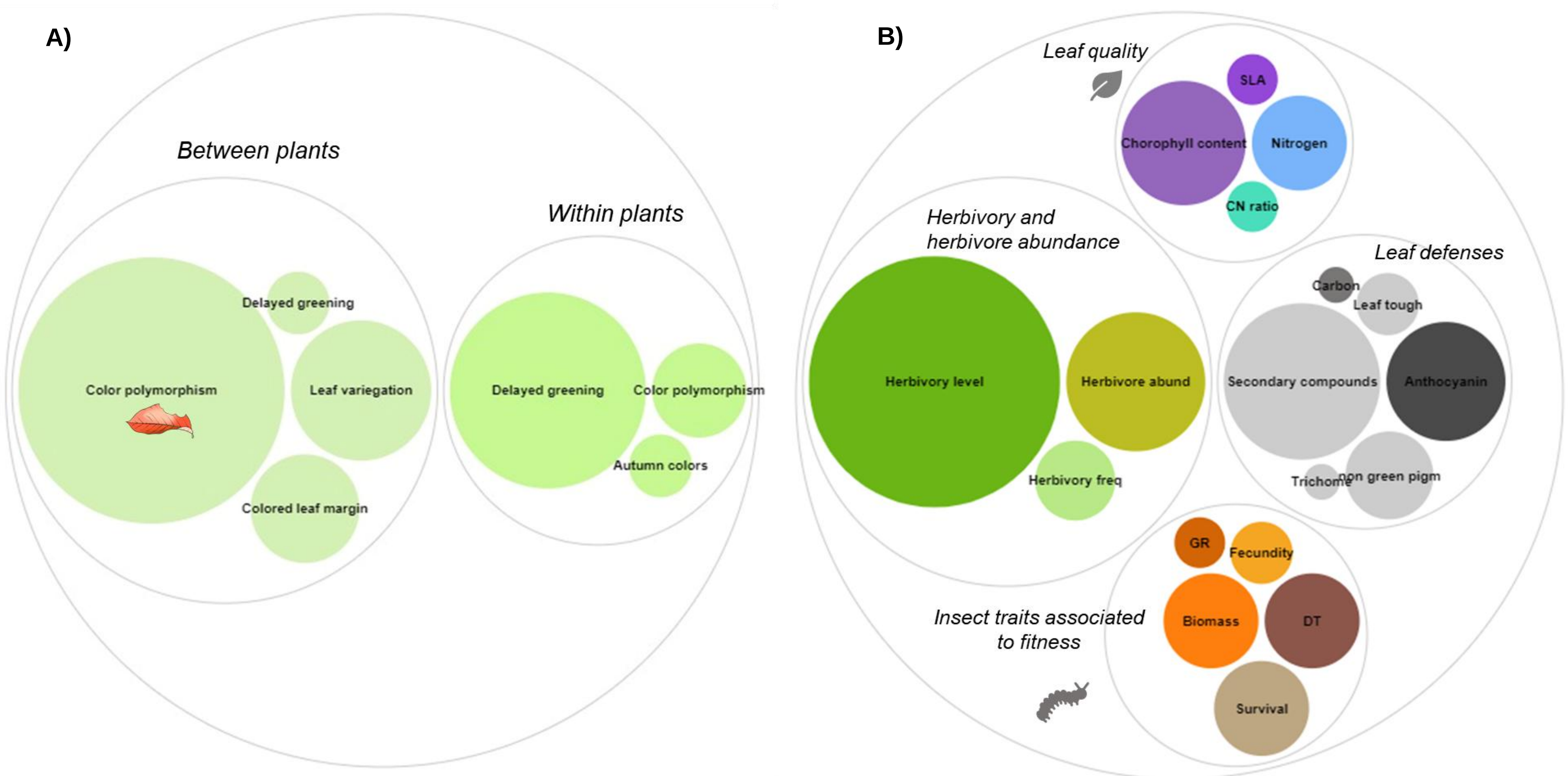

New Phytologist

Fig.S2
The aluvial plot shows the distribution and number of independent outcomes among categories of region, leaf color, herbivore guild and response variables used in the meta-analyses.

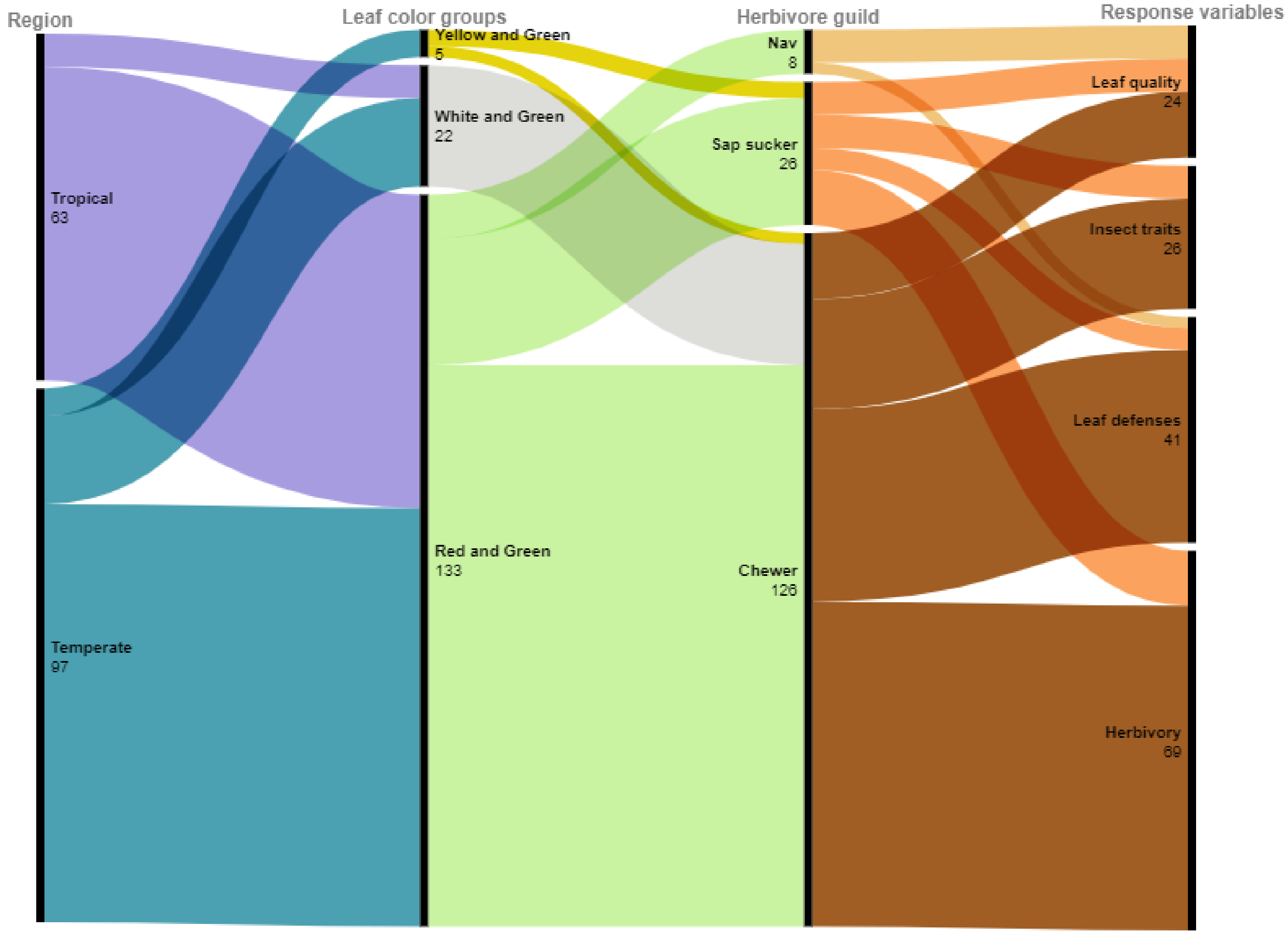

New Phytologist

Table S1. Assessment of publication bias in overall models for effects of leaf color on 4 response variables (Herbivory, Leaf defenses, Leaf nutritional quality and Insect traits associated to performance). Total Heterogeneity (QT) was significant in all cases; fail-safe numbers indicate the robustness of meta-analysis (larger then 5K+10, where k is the number of comparisons in each analysis); intercepts of Egger´s regression were not significantly different from zero, and Trim and fill analyses indicated a small number of articles to be added to analyses to turn funnel plots into symmetrical. (*P<0.05, **P<0.001)

Response Variable	Heterogeneity (Q_T)	Fail-safe Number	Egger´s intercept	I^2 (CI)	Trim and fill number
Herbivory	99.65**	95651	-0.45	79.89 (74.99 to 83.84)	2
Leaf defenses	97.80**	23071	0.62	69.17 (57.57 to 77.6)	11
Leaf quality	99.12**	59246	-0.319	72.05 (57.88 to 81.39)	6
Insect traits	96.88*	16256	-0.18	37.78 (0.11 to 61.24)	1

Fig S3 - Trim and fill funnel plots for each response variable used in the meta-analysis. Gray dots indicate the calculated effect sizes and red dots indicate the number of studies estimated by trim and fill to be included in the meta-analyses to turn plots of precision against effect size into symmetrical shape. The dashed gray line represents the mean effect size of the original data and the dashed red line shows the mean effect size including the inferred data. The low number of studies to be added by the trim and fill method (Herbivory: 2 studies, Leaf defenses: 11 studies, Leaf quality: 6 studies, Insect traits: 1 study) indicate the robustness of the meta-analyses.

HERBIVORY

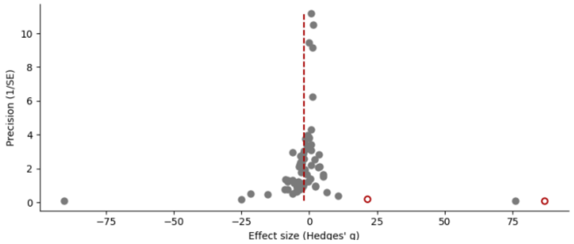

LEAF DEFENSES

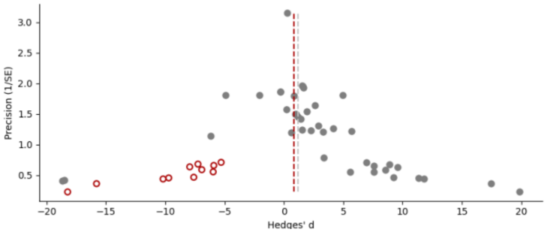

LEAF NUTRITIONAL QUALITY

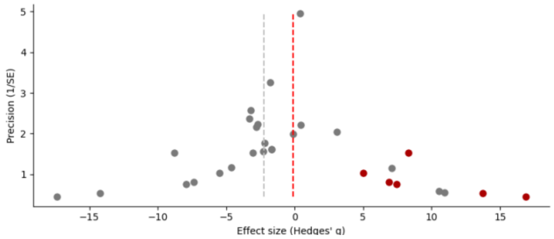

INSECT FITNESS

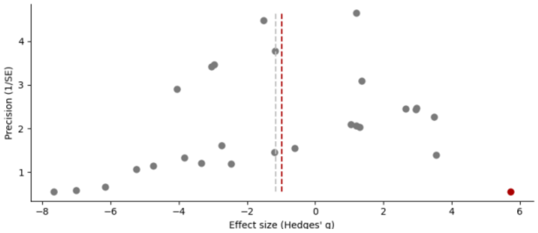

Notes S1
Qualitative results

Studies evaluating the intraspecific effects of leaf color on herbivory that met our inclusion criteria were conducted over the past 30 years in 18 different countries, in both tropical (12 studies, 63 effect sizes) and temperate regions (15 studies, 97 effect sizes). Twenty-seven studies of 47 plant species from 29 families resulted in a total of 160 effect sizes. Most effect sizes (59%) included paired comparison of green and red leaves, with fewer comparing green with purple (14.4%), white/silver (13.8%), red margins (7.5%), and yellow (3.1%). *In situ* studies were conducted in temperate forests (30.76%), tropical rainforests (26.9%) and other biomes such as dry-forests (11.53%) and tundra (3.84%), with approximately one fourth (26.9%) of the studies done in common gardens or greenhouses. Data are available for 47 plant species (Supplementary Material S4) distributed in 29 families, with Rosaceae, Lamiaceae, and Myrtaceae as the most common families in our database. Although the paired combination of red and green leaves was the most common (59% of the effect sizes), other color combinations were also reported in the literature (purple and green: 14.4%; white/silver and green 13.8%; red margins and green: 7.5%). Surprisingly, studies with yellow leaves were scarce, encompassing only 3.1% of the registers.

Several herbivores have been addressed in these studies, including caterpillars (41.3% of the effect sizes), grasshoppers (10.7%), beetles (7.5%), psyllids (8.8%), and aphids or mealybugs (7.5%). In 21% of the studies, the authors did not report the herbivore group. Most herbivores were chewers (76.3%) whereas sap-feeders encompassed only 16.3% of the registers. Herbivory was the most common response variable evaluated in the published studies, representing almost 45% of all the effect

sizes whereas herbivory intensity (the proportional amount of leaf tissue removed) was the most common measurement of insect damage in colored and green leaves. Evaluation of leaf defenses (25.6% of the comparisons) and leaf quality (15% of the comparisons) were also performed in red-green leaves through the quantification of secondary compounds or quantification of the amounts of nutrients and insect traits were evaluated in only six studies. None of the studies evaluated all four groups of response variables.

Table S2. Studies conducted at communities of plants in the tropics, evaluating herbivory and/or leaf traits at plants with exclusively young green or exclusively young red leaves. Comparisons in the primary data were made between species of plants with red or green leaves or between young leaves of plants with or without delayed greening.

Author and Year	Region	Locality	Response Variable	Effect Size	Number of species
Coley&Aide_1989	Tropical	Panama	Herbivory	-0.73	20
Chen&Huang_2013	Tropical	China	Leaf thickness	0.0022	76
Gong_etal_2020	Tropical	China	Herbivory	-0.141	250
Gong_etal_2020	Tropical	China	Anthocyanin	0.888	250
Gong_etal_2020	Tropical	China	Chlorophyll	-0.603	250
Gong_etal_2020	Tropical	China	Tannin	0.666	250
Martin_Eberhardt_2021	Tropical	Costa_Rica	Herbivory	-0.831	6
Martin_Eberhardt_2021	Tropical	Costa_Rica	Anthocyanin	2.4329	6

References

O'Dea RE, Lagisz M, Jennions MD, Koricheva J, Noble DW, Parker TH, Gurevitch J, Page MJ, Stewart G, Moher D, Nakagawa S. Preferred reporting items for systematic reviews and meta-analyses in ecology and evolutionary biology: a PRISMA extension. Biol Rev 2021;96(5):1695-1722. doi: 10.1111/brv.12721

Page MJ, McKenzie JE, Bossuyt PM, Boutron I, Hoffmann TC, Mulrow CD, et al. The PRISMA 2021 statement: an updated guideline for reporting systematic reviews. BMJ 2021;372:n71. doi: 10.1136/bmj.n7

Coley PD & Aide MT (1989) Red coloration of tropical young leaves: a possible anti-fungal defense? Journal of Tropical Ecology 5: 293-300.

Chen YZ & Huang SQ (2013) Red Young leaves have less mechanical defence than green youg leaves. Oikos 122: 1035-1041.

Gong W, Liu Y, Wang C, Chen Y, Martin K & Meng L (2020) Why are there so many plant species that transiently flush young leaves red in the tropics? Frontiers in Plant Science 11: 83.

New Phytologist

Martin-Eberhardt S (2021) Delayed greening and herbivory defense in Neotropical understory plants. Unpublished report.
